# Rumen mycobiome dynamics and dairy productivity: functional contributions of fungi to milk yield in bovine ruminants

**DOI:** 10.3389/ffunb.2026.1878257

**Published:** 2026-07-21

**Authors:** Gurpreet Singh, Lavika, Nandini, Abhay, Deepika, Nishchal Thakur, Robin Kaura, Sukhraj Kaur, Harvinder Singh Saini, Sagar Khulape, Anil Kumar Puniya, Harmeet Singh Dhillon

**Affiliations:** 1 Department of Microbiology, Guru Nanak Dev University, Amritsar, Punjab, India; 2 Dairy Microbiology Division, Indian Council of Agricultural Research (ICAR)-National Dairy Research Institute, Karnal, India; 3 Punjab Dairy Development Department, Mohali, Punjab, India; 4 Dairy Engineering Division, Indian Council of Agricultural Research (ICAR)-National Dairy Research Institute, Karnal, India; 5 Indian Council of Agricultural Research (ICAR)-National Research Centre on Camel, Bikaner, India

**Keywords:** dairy cattle, fiber degradation, milk productivity, *Neocallimastigomycota*, omics technology, rumen mycobiome

## Abstract

The rumen microbiome is a key factor influencing feed efficiency and milk production in ruminants. However, most studies have focused on the bacterial and archaeal components, with the fungal fraction of the rumen being relatively understudied. The unique ability of anaerobic rumen fungi of the phylum *Neocallimastigomycota* to colonize and degrade lignocellulosic biomass in the rumen through mechanical disruption of plant cell walls has now made them well known as important functional members of the rumen ecosystem. These fungi are armed with a plethora of carbohydrate-active enzymes degrading fiber and increase the availability of substrates for microbial fermentation. This leads to increased production of volatile fatty acids, especially acetate, which is a major precursor for milk fat production. Recent research evidence has indicated that rumen fungal communities are associated with feed efficiency, fermentation dynamics, and milk composition traits such as fat and protein content in bovine ruminants such as cattle and buffalo. However, most of the studies available are correlative, and the direct causal relationships between fungal activity and milk productivity are poorly delineated. High-throughput sequencing and multi-omics approaches have broadened our knowledge of fungal diversity and function; however, there are still limitations in reference databases and methodological biases. This review summarizes the current knowledge of the diversity, ecological roles, and functional contributions of anaerobic rumen fungi, with special reference to their association with milk production. It also highlights significant methodological and conceptual gaps and proposes future avenues for the integration of fungal ecology into microbiome-based approaches to improve dairy productivity. We need a holistic multi-kingdom view of the rumen microbiome to design efficient and sustainable dairy production systems.

## 1 Introduction

Dairy production is a major constituent of the global agriculture landscape, contributing significantly to nutritional security as well as the socioeconomic well-being of millions of farmers (Bhagat et al., 2023). Among ruminant animals, bovine species of cattle (*Bos taurus*) and buffalo (*Bubalus bubalis*) represent the principal milk producers, particularly in the South Asian region, where buffalo milk accounts for a substantial proportion of milk yields (Siddiky, 2017; de la Cruz-Cruz et al., 2026). Variations in milk yield and composition are governed by a complex interaction of factors, including the genetic makeup of the host, animal nutrition and management practices, as well as the microbial efficiency within the rumen ecosystem (Bickhart and Weimer, 2018). The rumen, the first tract of ruminants’ four-chambered stomach, hosts complex microbial communities, including bacteria, archaea, fungi, and protozoans, which are pivotal to digest plant material while providing the host with valuable fermentation end products (Bickhart and Weimer, 2018; de la Cruz-Cruz et al., 2026). Historically, rumen microbiology research has been disproportionately focused on bacterial and archaeal communities, largely due to methodological constraints and the widespread use of 16S rRNA-based approaches (McSweeney et al., 2006). Consequently, the fungal component of the rumen microbiome has remained comparatively underexplored (McSweeney et al., 2006; Bickhart and Weimer, 2018). Nevertheless, anaerobic rumen fungi belonging to the phylum *Neocallimastigomycota* are now recognized as integral members of the rumen ecosystem (McSweeney et al., 2006).

Microbial communities in the rumen interact among themselves to produce lignocellulolytic enzymes that aid in digestion and also yield volatile fatty acids like acetate, propionate, butyrate, and formic acids, along with CO_2_, H_2_, and CH_4_ (Choudhury et al., 2015). The recent and rapid advancement in molecular microbiology and genomic techniques has allowed new approaches that have made it possible to examine the structure and properties of microbiota and mycobiota residing in the rumen (McSweeney et al., 2006; Choudhury et al., 2015). Understanding rumen microbiology is aided by looking at each microbe’s function, structure, makeup, and diversity (Paswan et al., 2022). So, in light of these considerations, there is a pressing need to systematically evaluate the role of gut fungi within the broader rumen microbiome framework and their contribution to dairy productivity (Choudhury et al., 2015; Paswan et al., 2022). Therefore, the present review aims to (i) synthesize current knowledge on the diversity and ecological roles of anaerobic rumen fungi, (ii) elucidate their functional contributions to fiber degradation and fermentation processes, (iii) critically assess existing evidence linking fungal communities to milk yield and composition in cattle and buffalo, and (iv) identify key knowledge gaps and future research directions. By integrating emerging insights from the rumen mycobiome research, this review seeks to advance a more holistic understanding of microbial determinants of milk productivity for sustainable dairy production systems.

## 2 Rumen ecosystem and microbial interactions

### 2.1 Composition of the rumen microbiota

The rumen is a specialized compartment of the ruminant’s four-chambered stomach and contains a highly complex and dynamic microbial ecosystem that consists of protozoa, bacteriophages, anaerobic bacteria, anaerobic rumen fungi, and methanogenic archaea (Choudhury et al., 2015; Paswan et al., 2022). The microbial consortia carry out the degradation and fermentation of carbohydrates and proteins to allow the host animal to efficiently utilize fibrous plant-based diets (Bickhart and Weimer, 2018; Paswan et al., 2022). Bacteria are the most numerous and functionally diverse members of the rumen microbiota of these populations, and occur at densities of approximately 10^12^ cells per gram of rumen content (Paswan et al., 2022; de la Cruz-Cruz et al., 2026). Their metabolic versatility is due to the presence of a wide variety of enzymes that can degrade plant cell components like cellulose, hemicellulose, lignin, starch, proteins, and lipids (Choudhury et al., 2015; Paswan et al., 2022). The major bacterial phyla are *Bacillota* (earlier called *Firmicutes)*, *Bacteroidota* (earlier called *Bacteroidetes)*, and *Pseudomonadota* (earlier called *Proteobacteria)*, which have a major role in ruminal digestion and fermentation (Costa-Roura et al., 2022; Paswan et al., 2022). Methanogenic archaea are a specialized group of functional rumen organisms that are generally present at 10^6^ to 10^8^ cells per milliliter of rumen content (Costa-Roura et al., 2022; Paswan et al., 2022). Most of these microbes are members of the *Methanobacteriaceae* family. These microorganisms perform hydrogenotrophic methanogenesis, i.e., use of hydrogen (H_2_) produced by bacterial fermentation for the reduction of carbon dioxide (CO_2_) to methane (CH_4_). The process is important not only for the redox balance in the rumen, but also contributes to enteric methane emissions (Costa-Roura et al., 2022; Keum et al., 2024).

Additional important players in plant food digestion in the rumen ecosystem are anaerobic rumen fungi, with a high lignocellulolytic potential (Elghandour et al., 2020; Agustina et al., 2022). Anaerobic rumen fungi play an important role in fiber degradation by secretion of a range of enzymes, including cellulases, hemicellulases, and lignin-modifying enzymes, which results in improved nutrient availability and digestibility (Elghandour et al., 2020; Agustina et al., 2022). Anaerobic rumen fungi are a functionally important component of the rumen ecosystem, but are less abundant than bacteria, usually in the range of 10³–10^6^ cells/ml of rumen content (Elghandour et al., 2020; Keum et al., 2024). These fungi are proteolytic and saccharolytic and are involved in the physical and enzymatic degradation of plant fibers (Summers and Arfken, 2022). Moreover, within the rumen ecosystem, ciliate protozoa and viruses function as critical “predators within” that profoundly shape microbial community dynamics and host metabolic efficiency (Yu et al., 2024a). Protozoa in the rumen potentially alter the microbiome through top-down predation and bottom-up carbohydrate sequestration, yet they also drive wasteful intraruminal microbial protein recycling and promote methanogenesis (Yu et al., 2024a; Yu et al., 2025). Similarly, a large number of rumen viruses regulate host populations via lytic cycles, further intensifying protein recycling while utilizing auxiliary metabolic genes (AMGs) during lysogeny to augment microbial metabolic fitness (Yu et al., 2024a; Yu et al., 2025). Gaining a dynamic understanding of these complex predator-prey dynamics offers innovative, targeted pathways to mitigate methane emissions, prevent metabolic disorders like acidosis, and enhance nitrogen utilization efficiency in sustainable livestock production (Yu et al., 2024a; Yu et al., 2025). Furthermore, strong associations among ruminal microbial composition, volatile fatty acid (VFA) profiles, and milk composition are reported in several studies (Agustina et al., 2022; Keum et al., 2024). Acetate, propionate, and butyrate are the major VFAs, and they are positively correlated with total VFA concentration (Yu et al., 2024a; Yu et al., 2025). These are the primary end products of microbial fermentation of structural and non-structural carbohydrates like cellulose, hemicellulose, and starch (Gruninger et al., 2014; Keum et al., 2024). These short-chain fatty acids are important metabolic precursors, acetate and butyrate being especially important for milk fat synthesis and propionate being a major gluconeogenic substrate for lactose synthesis and indirectly for milk protein synthesis (Iqbal et al., 2018; Cox et al., 2021).

### 2.2 Ecological role of fungi in the rumen microbiota

Anaerobic rumen fungi (ARF) belong to the phylum *Neocallimastigomycota* that are found throughout the gastrointestinal tract of ruminants and represent a key functional group in fiber degradation (Ji et al., 2022; Keum et al., 2024). ARF greatly improves the accessibility of plant cell wall constituents by mechanical disruption (rhizoidal penetration) and enzymatic hydrolysis, resulting in better overall ruminal fiber digestion (Elghandour et al., 2020; Ji et al., 2022; Yu et al., 2024a). Anaerobic rumen fungi are metabolically active and produce volatile fatty acids (VFAs), mainly acetate. VFAs play an important role in energy metabolism and serve as a precursor for milk fat synthesis (Gruninger et al., 2014; Iqbal et al., 2018). Besides their involvement in fermentation, ARF are also involved in the microbial protein pool, thus supporting the host nutrition (Iqbal et al., 2018; Ji et al., 2022). ARF is known to play an ecological role in the rumen, and studies have reported that supplementation or higher abundance of ARF is associated with better growth performance in calves and higher milk production in lactating buffaloes (Kumar et al., 2015; Ji et al., 2022). Some ARF taxa, such as *Orpinomyces* spp., are also involved in biohydrogenation of polyunsaturated fatty acids (PUFAs) like linoleic acid (LA) into conjugated linoleic acids (CLAs) (Cheng et al., 2018; Summers and Arfken, 2022). These bioactive lipids have been linked to a multitude of health benefits in ruminants, including the modulation of immune function and a decrease in the occurrence of metabolic disorders such as hyperketonemia in early lactation (Cheng et al., 2018; Hartinger and Zebeli, 2021; Swift et al., 2021). In addition, CLAs in products from ruminants are considered to be good for human health, and research has suggested that they may have anti-carcinogenic effects (Matthews et al., 2019; Hartinger and Zebeli, 2021).

## 3 Diversity and taxonomy of gut fungi

### 3.1 Taxonomic overview of anaerobic rumen fungi

Anaerobic rumen fungi (ARF) are mainly found in the rumen of herbivorous ruminants and grow in strictly anaerobic conditions (Matthews et al., 2019). ARF belongs to the phylum *Neocallimastigomycota*, order *Neocallimastigales*, and family *Neocallimastigaceae*. This phylum currently comprises 36 described species in 20 genera (Paul et al., 2018; Matthews et al., 2019; Hanafy et al., 2022). Well-characterized genera include *Neocallimastix*, *Piromyces*, *Orpinomyces*, *Anaeromyces*, *Caecomyces*, and *Cyllamyces* (Paul et al., 2018; Hanafy et al., 2022). However, the taxonomic resolution of several additional genera remains uncertain due to the lack of cultured representatives and limited molecular characterization, indicating the need for further phylogenetic and genomic investigations (Paul et al., 2018; Hanafy et al., 2022).

### 3.2 Morphological and functional diversity

Classification of anaerobic rumen fungi (ARF) has traditionally been based on the morphology of the thallus and the architecture of the rhizoids (Paul et al., 2018; Matthews et al., 2019; Hanafy et al., 2022). ARF are usually classified as monocentric or polycentric according to the thallus organization (Matthews et al., 2019; Hanafy et al., 2022). For example, *Neocallimastix* has a monocentric thallus (one sporangium per thallus) while *Anaeromyces* has a polycentric growth form (many sporangia growing from an elaborate network of rhizoids) (Paul et al., 2018). Rhizoidal morphology and zoospore characters provide further taxonomic distinction (Paul et al., 2018). *Neocallimastix frontalis* has well-developed filamentous rhizoids and releases poly-flagellated zoospores, which improves the efficiency of the fungus to colonize fibrous substrates (Hanafy et al., 2022). The anaerobic rumen fungi are broadly divided into two distinct groups based on how they physically target and break down plant tissue (Paul et al., 2018). These include the filamentous (rhizoid-forming) genera, including *Neocallimastix*, *Piromyces*, and *Anaeromyces*. These fungi develop long, deeply branching rhizoidal networks (similar to fungal hyphae) that physically invade and penetrate deep into tough, recalcitrant plant tissues (Paul et al., 2018; Hanafy et al., 2022). Because they locate the vast majority of their plant-cell-wall-dismantling enzymes right at these rhizoids, they have a distinct advantage in decomposing highly lignified tissues (Paul et al., 2018). The other group includes bulbous genera that contain *Caecomyces* and *Cyllamyces* genera (Wang et al., 2020). These forms do not produce long, penetrating rhizoids. Instead, their sporangia launch with very short extensions, and they attach themselves directly to the surface of the plant material. These ARF rely on mechanical breakdown that has already occurred (e.g., fodder that has already been chewed by the host animal), preferring physically fragmented, surface-exposed tissues (Paul et al., 2018; Wang et al., 2020). For example, *Caecomyces churrovis* produces substantial amounts of free enzymes capable of degrading plant cell wall polymers, thus compensating for the absence of mechanical penetration (Henske et al., 2017). This functional adaptation illustrates the metabolic diversity among ARF taxa (Solomon et al., 2016).

Across all these genera, the ultimate tool for executing biomass degradation is a cellulosome, which is a highly organized, surface-bound multienzyme complex (Paul et al., 2018; Wang et al., 2020). Rather than just secreting free-floating enzymes, genera like *Neocallimastix* and *Piromyces* coordinate their CAZymes into these complexes using dockerin, cohesin, and scaffoldin proteins (Henske et al., 2017; Wang et al., 2020). This architecture anchors non-catalytic Carbohydrate-Binding Modules (CBMs) to the plant surface, allowing the coordinated Glycoside Hydrolases to degrade the substrate up to 50-fold more efficiently than independent, secreted enzymes (Wang et al., 2020). For example, *Neocallimastix (N. cameronii* variants) are extensively rich in carbohydrate-active genes and carry between 2,480 and 3,452 total CAZymes across their sequenced strains, including glycoside hydrolases, carbohydrate esterases, polysaccharide lyases, and dockerins that are essential for assembling their multi-enzyme complexes) (Kameshwar and Qin, 2018; Yücel and Ekinci, 2022; Kovács et al., 2025). *Piromyces* (*Piromyces* sp. E2 and *P. finnis*) reportedly possesses between 1,385 and 1,894 total CAZymes, including 278 to 472 glycoside hydrolases (GH) and 32 to 39 polysaccharide lyases (PL) (Kameshwar and Qin, 2018; Yücel and Ekinci, 2022; Kovács et al., 2025). *Caecomyces* (*C. communis*) also contains 1,984 total CAZymes, including 303 glycoside hydrolases (GH), 83 carbohydrate esterases (CE), and 8 polysaccharide lyases (PL). *Orpinomyces* (*Orpinomyces* sp.) has 1,778 total CAZymes (Kameshwar and Qin, 2018; Yücel and Ekinci, 2022; Kovács et al., 2025). *Anaeromyces* (*A. robustus*) encodes 1,609 total CAZymes, which include 261 glycoside hydrolases (GH), 11 polysaccharide lyases (PL), and some 15 CBM87 anchoring modules (Kameshwar and Qin, 2018; Yücel and Ekinci, 2022; Kovács et al., 2025).

### 3.3 Environmental and dietary influences on fungal diversity

The dominant ARF genera in ruminants are *Piromyces*, *Caecomyces*, and *Neocallimastix* (Paul et al., 2018; Wang et al., 2020; Hanafy et al., 2022). These fungi are among the first colonizers of ingested plant material and initiate fiber degradation by mechanical disruption through rhizoidal penetration and subsequent enzymatic hydrolysis (Solomon et al., 2016; Henske et al., 2017). ARF are a permanent feature of ruminants and have conserved functional roles, but their relative abundance varies with host species, diet, and environmental or physiological conditions (Henske et al., 2017; Kameshwar and Qin, 2018; Kovács et al., 2025). Host physiology and dietary transitions strongly influence the composition of the rumen anaerobic fungal community (Kameshwar and Qin, 2018; Yücel and Ekinci, 2022). For example, the transition from the dry period to early lactation of dairy cows is associated with large changes in rumen microbial ecology, including perturbations in fungal diversity and community structure (Tripathi et al., 2007). Similarly, environmental stressors such as heat stress can disrupt the overall rumen microbiota, but anaerobic rumen fungi (ARF) are often relatively resilient and able to maintain functional activity under such stress conditions (Thongbunrod and Chaiprasert, 2026).

**Figure 1:**
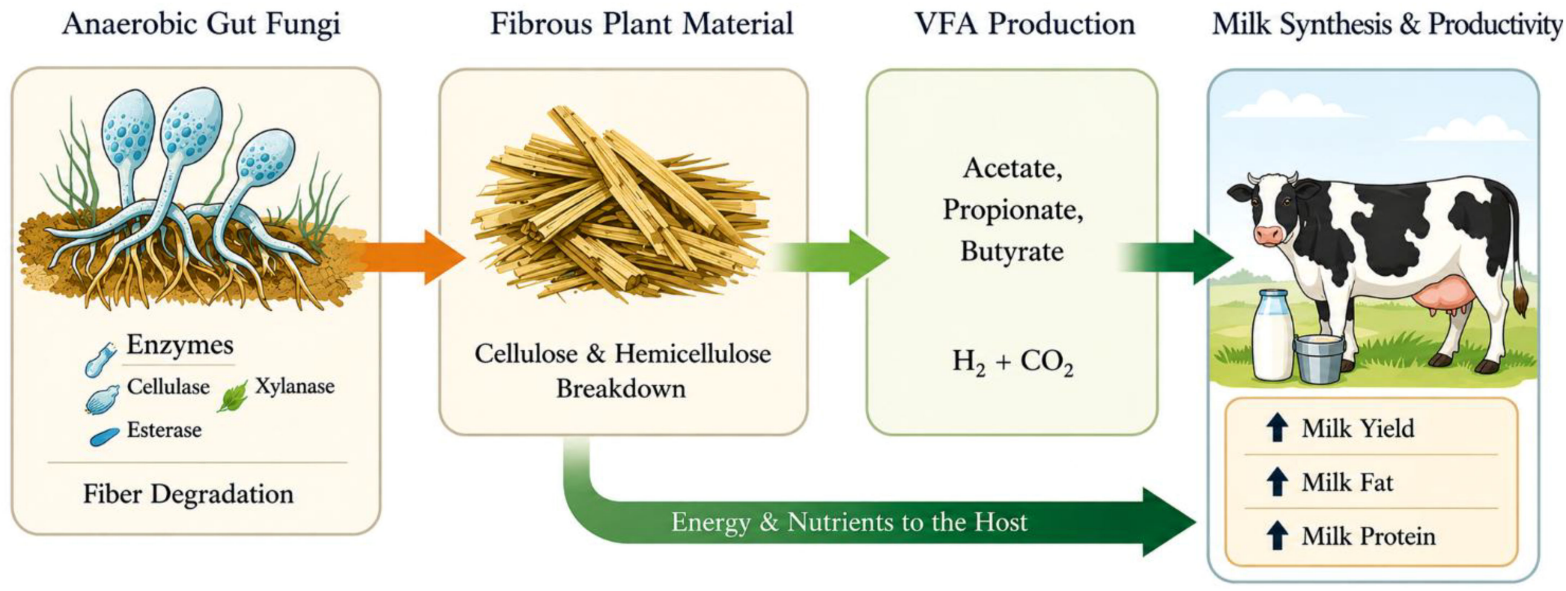
Anaerobic rumen fungi degrade fibrous plant material by means of fibrolytic enzymes and produce volatile fatty acids along with metabolic gases. These fermentation products are energy and nutrients for the host and increase milk yield, milk fat, and milk protein production.

Dietary composition is important in shaping ARF communities. High-grain diets lower rumen pH and suppress some microbial populations, but ARF can maintain fibrolytic activity by colonizing plant material and releasing potent lignocellulolytic enzymes (Edwards et al., 2017; Kovács et al., 2025). Their persistence and functionality in mildly acidic conditions enable them to support fiber degradation even during dietary regimens less favorable to classical fibrolytic bacteria. Interestingly, these fungi exhibit broadly similar classes of lignocellulolytic enzymes, but there is inter-genus variability in the ability to secrete enzymes and growth strategies, which translates to differences in degradation efficiency and ecological adaptation (Tovar-Herrera et al., 2026). Fungal diversity in the rumen is closely associated with host productivity as ARF have a strong impact on fibrolytic activity, volatile fatty acid (VFA) profiles, and pathways of milk synthesis (**Figure 1**). The acetate produced during fungal-driven fermentation is a particularly important precursor for milk fat synthesis (Edwards et al., 2017; Tovar-Herrera et al., 2026). Fungal biomass also contributes to the microbial protein pool and contains the essential amino acid lysine, which is critical to casein synthesis and overall milk protein yield (Jyothi et al., 2024; Tovar-Herrera et al., 2026).

### 3.4 Host-specific variations: cattle vs buffalo

The composition and distribution of anaerobic rumen fungi (ARF) are highly variable and are strongly influenced by host species, dietary regime, digestive physiology, and environmental conditions (Deng et al., 2025). Differential host-specific patterns reported between buffalo (*Bubalus bubalis*) and cattle (*Bos taurus*) are a reflection of differences in rumen ecology and feed utilization strategies (Tripathi et al., 2007; Yücel and Ekinci, 2022; Deng et al., 2025; Tovar-Herrera et al., 2026). Polycentric fungal taxa like *Orpinomyces* are dominant in *Bubalus bubalis* and hold high potential for degradation of recalcitrant lignocellulosic substrates (Jyothi et al., 2024; Deng et al., 2025). Their rhizoidal networks are extensive, penetrating deeply into the plant tissues, and breaking down rigid cell wall structures (Deng et al., 2025). This functional trait is consistent with the ability of the buffalo to utilize high-lignin roughages efficiently. Also, the buffalo rumen contains monocentric taxa like *Neocallimastix frontalis* and members of the genus *Caecomyces* that are also common in fiber degradation (Jyothi et al., 2024; Deng et al., 2025; Tovar-Herrera et al., 2026). In contrast, the *Bos taurus* rumen is often dominated by monocentric, rhizoidal fungi such as *Neocallimastix*, which have fast growth rates and a significant fermentative capacity (Jyothi et al., 2024; Deng et al., 2025; Tovar-Herrera et al., 2026). These fungi are reported to generate large multi-enzyme complexes, such as cellulosomes with high lignocellulolytic activity (Jyothi et al., 2024; Deng et al., 2025; Tovar-Herrera et al., 2026). The fermentation end-products produced by *Neocallimastix* spp. are higher than those produced by the genera *Piromyces* and *Caecomyces*, which may increase energy availability and are associated with an increase in milk fat synthesis (Choudhury et al., 2015). Comparative studies among bovine species have also highlighted physiological and microbiological differences influencing feed utilization efficiency. Buffaloes have better fermentation efficiency than cattle under similar feeding and management systems, mainly due to better utilization of fermentable substrates (Choudhury et al., 2015; Kumar et al., 2015). This improvement in efficiency has been partially attributed to a relatively higher population of anaerobic fungal zoospores in the rumen that contribute to increased fiber degradation (Choudhury et al., 2015; Kumar et al., 2015). Furthermore, buffaloes have a superior nitrogen recycling capacity and ruminate about 50% more than cattle, which together help to enhance digestion and nutrient assimilation (Kumar et al., 2015).

## 4 Functional role of gut fungi in the rumen

### 4.1 Fibre degradation and plant cell wall disruption

Anaerobic rumen fungi (ARF) belonging to the phylum *Neocallimastigomycota* are primary early colonizers of lignocellulosic biomass in the rumen and are essential for the initiation of fiber degradation (Bickhart and Weimer, 2018; Costa-Roura et al., 2022). They accomplish this by producing highly active carbohydrate-active enzymes (CAZymes) and multienzyme complexes, termed cellulosomes, that synergistically improve fibrolytic efficiency (Gruninger et al., 2014; Swift et al., 2021; Yu et al., 2025). The enzymatic machinery of ARF is composed of cellulases, including endoglucanases and exoglucanases that act synergistically to hydrolyze cellulose to soluble oligosaccharides and glucose (Henske et al., 2017; Wang et al., 2020; Deng et al., 2025). Meanwhile, hemicellulose degradation is catalyzed by enzymes such as endo-xylanases, β-xylosidases (xylobiose), and acetyl xylan esterases, which allow the breakdown of complex hemicellulosic polymers into fermentable sugars (Deng et al., 2025). The large and diverse CAZyme profile of Anaerobic rumen fungi is suggestive of their specialization in lignocellulose degradation. Genomic and transcriptomic analyses identified a large diversity of enzyme families, including numerous glycoside hydrolases (GHs) and polysaccharide lyases (PLs), that work collectively to break down plant cell wall architecture (Wilken et al., 2021).

While the Cellulase activity is extensively carried out via glycoside hydrolase enzyme classes GH-1, GH-2, GH-3, GH-5, GH-6, GH-8, GH-9, GH-38, GH-45, GH-48, and GH-74 (Kameshwar and Qin, 2018; Yücel and Ekinci, 2022; Kovács et al., 2025). *Neocallimastix* (*N. californiae*) is particularly studied for having the highest predicted cellulolytic activity, while *Anaeromyces* exhibits comparatively lower activity among ARF (Kameshwar and Qin, 2018; Yücel and Ekinci, 2022; Kovács et al., 2025). Similarly, hemicellulose is manly hydrolyzed by classes GH-10, GH-11, GH-30, GH-31, GH-38, GH-39, GH-43, GH-45, GH-47, GH-53, GH-115, alongside carbohydrate esterase classes CE-1, CE-2, CE-3, CE-4, CE-6, and CE-16 (Kameshwar and Qin, 2018; Yücel and Ekinci, 2022; Kovács et al., 2025). *Piromyces finnis* exhibits lower relative hemicellulolytic activity, whereas *Neocallimastix* exhibits the highest hemicellulase activity (Kameshwar and Qin, 2018; Yücel and Ekinci, 2022; Kovács et al., 2025). Pectinolytic enzymes are involved in pectin degradation using polysaccharide lyases (PL-1, PL-3, PL-4, PL-9, PL-11), glycoside hydrolases (GH-28, GH-78, GH-95, GH-105, GH-115), and carbohydrate esterases (CE-8, CE-12, and CE-16) (Kameshwar and Qin, 2018; Yücel and Ekinci, 2022; Kovács et al., 2025). Moreover, the genomes of these anaerobic rumen fungi are uniquely packed with carbohydrate-binding modules (CBMs) and dockerin proteins (specifically cellulose binding family 5) (Kameshwar and Qin, 2018; Yücel and Ekinci, 2022; Kovács et al., 2025). These modules assemble into large multi-protein complexes called cellulosomes, which mechanically adhere to and efficiently hydrolyze plant biomass (Kameshwar and Qin, 2018; Yücel and Ekinci, 2022; Kovács et al., 2025).

### 4.2 Fermentation and metabolic production

Anaerobic rumen fungi (ARF) generate predominantly formate, butyrate, acetate, H_2,_ and CO_2_ as end products of fermentation, which collectively contribute to rumen energy metabolism (Agustina et al., 2022; Costa-Roura et al., 2022). Of these, acetate is an important metabolite and a primary precursor for *de novo* fatty acid synthesis in bovine mammary epithelial cells (Wilken et al., 2021; Lin et al., 2022). Acetate has been reported to enhance lipid biosynthesis through activation of the mTOR signaling pathway, resulting in increased triglycerides and milk fat yield in dairy animals (Wilken et al., 2021; Lin et al., 2022). Metabolically, species such as *Neocallimastix lanati* have well-developed pathways for amino acid biosynthesis (e.g., arginine) and lipid metabolism (Wilken et al., 2021; Lin et al., 2022). There have been two major pathways described for pyruvate metabolism in ARF: the pyruvate formate lyase (PFL) pathway and the pyruvate: ferredoxin oxidoreductase (PFO) pathway (Wilken et al., 2021; Lin et al., 2022). The PFL pathway was probably acquired by horizontal transfer from bacteria and converts pyruvate to formate and acetyl-CoA. Under certain conditions, this pathway is often more metabolically active than the PFO pathway (Wilken et al., 2021; Lin et al., 2022). On the other hand, the PFO pathway produces acetyl-CoA and reduced ferredoxin, and this results in hydrogen production by hydrogenosomes. Both pathways operate in specialized organelles, hydrogenosomes, which generate energy as ATP in the absence of oxygen (Wilken et al., 2021; Lin et al., 2022). The presence of these metabolic pathways enables ARF functional flexibility to adapt to the different redox conditions in the rumen and to maintain a high fermentative efficiency (Wilken et al., 2021; Lin et al., 2022).

ARF inhabit highly anoxic environments, and their fermentation pathways are uniquely adapted to maximize ATP (energy) yield in the absence of oxygen (Wilken et al., 2021; Lin et al., 2022). This includes the classical cytosolic pathway (glycolysis), which starts in the cytosol and involves the breakdown of complex plant polymers (cellulose and hemicellulose) into hexoses (glucose) and pentoses, which are then metabolized via standard glycolysis (the Embden-Meyerhof-Parnas pathway) (Kazda et al., 2014; Lin et al., 2022; Bhagat et al., 2023). Glycolysis is the conversion of glucose to pyruvate, with the reduction of NAD to NADH and a net gain of 2 ATP. In the cytosol, pyruvate can be directed into different end products depending on the fungal species and environmental conditions (Kazda et al., 2014; Wilken et al., 2021; Lin et al., 2022). These pathways can be lactate production, where pyruvate is reduced to lactate by lactate-dehydrogenase to regenerate NAD; succinate production, where pyruvate is carboxylated to malate, which then enters a partial, reversed TCA cycle pathway to produce succinate; or ethanol production, where pyruvate is converted to acetaldehyde and then reduced to ethanol to balance electron carriers (NADH) (Kazda et al., 2014; Bhagat et al., 2023). Also, a large amount of cytosolic pyruvate (or malate) is exported to the hydrogenosome to extract more energy from the remaining carbon (Kazda et al., 2014; Bhagat et al., 2023). Here, the peculiarities of anaerobic fungal fermentation are described (Kazda et al., 2014). Pyruvate is converted to acetyl-CoA and carbon dioxide in hydrogenosome (Kazda et al., 2014; Bhagat et al., 2023). This reaction is catalyzed by the enzyme pyruvate ferredoxin oxidoreductase (PFOR), which transfers electrons to a protein called ferredoxin, reducing it. Finally, a Fe-Fe hydrogenase enzyme re-oxidizes the reduced ferredoxin. This enzyme directs the electrons to protons, forming molecular hydrogen. PFOR then produces acetyl-CoA which is then converted to acetate (a major fermentation end product). This conversion is related to the conversion of ADP to ATP through the succinyl-CoA-synthetase/acetate SH-CoA-transferase enzyme loop (Kazda et al., 2014; Bhagat et al., 2023). The rumen methanogens are physically closely associated with the extracellular polysaccharide (EPS) biofilm matrix around the fungi. The continual removal of hydrogen acts as an electron sink, allowing the fungal fermentation pathway to run efficiently and constantly degrade plant material (Kazda et al., 2014; Bhagat et al., 2023).

### 4.3 Synergistic interactions with other rumen microorganisms

Anaerobic rumen fungi are the “icebreakers” of the rumen ecosystem, physically breaking down complex plant structural tissues with their rhizoidal networks (Kazda et al., 2014; Lin et al., 2022; Bhagat et al., 2023). But the mixed-acid fermentation occurring within their hydrogenosomes produces large quantities of molecular hydrogen and formate. In a natural ecosystem, the build-up of hydrogen ions in the environment can thermodynamically inhibit the fungal hydrogenase enzymes, slowing fermentation (Kazda et al., 2014; Bhagat et al., 2023). The fungi rely on a tightly integrated network of hydrogen-metabolizing bacteria, archaea, and methanotrophs to process these metabolic by-products and remain energetically efficient (Kazda et al., 2014; Lin et al., 2022; Bhagat et al., 2023). To prevent this, anaerobic rumen fungi are in close syntropic alignment with methanogenic archaea. The fungal hydrogen and carbon dioxide are consumed rapidly by the methanogens to produce methane (Kazda et al., 2014; Bhagat et al., 2023). Continuous removal of hydrogen keeps a low local partial pressure, shifting the fungal pathway to the more energy-efficient hydrogenosomal acetate pathway, optimizing the degradation of the recalcitrant fiber residues (Kazda et al., 2014; Lin et al., 2022; Bhagat et al., 2023).

In fungal hydrogenosomes, excess electrons are transferred onto protons by hydrogenase enzymes during fiber digestion, generating hydrogen (Kazda et al., 2014; Greening et al., 2019; Bhagat et al., 2023; McAllister et al., 2025). This accumulation of hydrogen builds up the partial pressure of hydrogen, which thermodynamically chokes the fungal hydrogenases, forcing the fungi to move away from the high-yielding acetate pathways to less energy-efficient end products such as ethanol or lactate (Kazda et al., 2014; Greening et al., 2019; Bhagat et al., 2023; McAllister et al., 2025). Anaerobic rumen fungi have developed close physical and chemical partnerships with two major groups of hydrogen-users, the methanogenic archaea (as the main electron sink) and the hydrogenotrophic and capnophilic bacteria (the competitors), to overcome this metabolic bottleneck (Kazda et al., 2014; Greening et al., 2019; Bhagat et al., 2023; McAllister et al., 2025). The best-known syntrophic partners of anaerobic rumen fungi are methanogens (e.g., *Methanobrevibacter ruminantium*), which attach physically to the fungal cell walls and extracellular structures, forming a dense biofilm (Kazda et al., 2014; Greening et al., 2019; Bhagat et al., 2023; McAllister et al., 2025). The methanogens consume hydrogen and formate from the fungus rapidly and reduce carbon dioxide to methane. This interspecies hydrogen transfer maintains a very low local hydrogen partial pressure, which strongly stimulates the fungus to increase its carbohydrate metabolism and enzyme secretion (Kazda et al., 2014; Greening et al., 2019; Bhagat et al., 2023; McAllister et al., 2025). Additionally, a number of true bacterial groups compete or cooperate in this hydrogen pool. The capnophilic (carbon dioxide-loving) and hydrogen-consuming bacteria such as *Fibrobacter succinogenes*, *Prevotella spp*, and *Ruminococcus flavefaciens* utilize hydrogen and carbon dioxide to form succinate or propionate (Kazda et al., 2014; Greening et al., 2019; Bhagat et al., 2023; McAllister et al., 2025). Moreover, unique sulfate/nitrate respirating bacteria like *Wolinella succinogenes* or *Denitrobacterium detoxificans* also play an alternative electron sink role by using hydrogen to reduce sulphur or nitrogen compounds (Kazda et al., 2014; Greening et al., 2019; Bhagat et al., 2023; McAllister et al., 2025). By constantly draining the hydrogen cloud around the fungal rhizoid, these bacteria keep the thermodynamic gradient available so that the fungal glycolysis and hydrogenosome function can continue unimpeded (Kazda et al., 2014; Greening et al., 2019; Bhagat et al., 2023; McAllister et al., 2025). Classically, well-studied methanotrophs are aerobic (require oxygen in order to carry out the first step of methane oxidation by the methane monooxygenase enzyme) (Kazda et al., 2014; Greening et al., 2019; Bhagat et al., 2023; McAllister et al., 2025). The rumen has long been considered to be strictly anoxic, implying that conventional aerobic methanotrophs could play only a minimal or no active role, limited to the micro-oxic microenvironments adjacent to the rumen wall (where a small amount of oxygen diffuses from the bloodstream) (Kazda et al., 2014; Greening et al., 2019; Bhagat et al., 2023; McAllister et al., 2025). In the fully anoxic rumen core, where the anaerobic rumen fungi thrive, methane oxidation is stimulated by specialized consortia that can perform Anaerobic Oxidation of Methane (AOM) (Kazda et al., 2014; Greening et al., 2019; Bhagat et al., 2023; McAllister et al., 2025). These are usually syntrophic associations of anaerobic methanotrophic archaea with sulfate-reducing or nitrate-reducing bacteria. Without hydrogen-metabolizing bacteria and methanogens to clean up the environment, the potent fiber-degrading pathways of anaerobic rumen fungi would grind to a halt due to metabolic self-poisoning (Kazda et al., 2014; Greening et al., 2019; McAllister et al., 2025).

### 4.4 Implications for milk production, nutrition, and composition

Anaerobic rumen fungi (ARF) are involved in the release of fermentable carbohydrates from lignocellulosic biomass via fibrolytic activity, which results in an increase in total digestible nutrients (TDN) and improvement of the overall feed efficiency (Wilken et al., 2021; Jyothi et al., 2024; Deng et al., 2025). These sugars are fermented by the microbes to produce volatile fatty acids (VFAs), mainly acetate, which is a major precursor for *de novo* fatty acid synthesis in the mammary gland and thus contributes greatly to the production of milk fat (Wilken et al., 2021). The ARF-derived enzymes exhibit excellent lignocellulolytic potential and are being increasingly explored for the development of industrial enzyme cocktails for biomass conversion (Wilken et al., 2021; Lin et al., 2022). In practice, rumen fungi are also used as feed additives for improving the nutritional value and digestibility of silage (Cann et al., 2025). The hydrolysis of plant polymers efficiently improves fiber digestibility and allows further fermentation to generate VFAs (acetate, propionate, and butyrate) and gases like hydrogen and carbon dioxide (Dhakal et al., 2025). These VFAs are absorbed through the rumen epithelium and used in host metabolism to contribute to energy supply, and indirectly to milk protein synthesis through provision of substrates for gluconeogenesis and microbial protein production (Wilken et al., 2021; Cann et al., 2025; Dhakal et al., 2025).

## 5 Link between gut fungi and milk productivity

### 5.1 Association between anaerobic rumen fungi and milk yield

The breakdown of fiber by anaerobic rumen fungi is an important determinant of how much they affect milk production (Li et al., 2018). Improved fibrolytic activity results in more efficient breakdown of plant cell wall components with increased release of fermentable substrates and fermentation efficiency (Li et al., 2018). This in turn stimulates higher production of volatile fatty acids (VFA) that are essential for energy metabolism and lactation performance. Recent large-scale studies of the rumen microbiome have associated lactation dynamics with the structure of the fungal community (Saxena et al., 2010; Li et al., 2018). In one extensive study, some anaerobic fungal populations showed strong correlations with production efficiency at various phases of lactation (Saxena et al., 2010; Meyer et al., 2019). Genera such as *Neocallimastix* and *Caecomyces* (family *Neocallimastigaceae*) were more abundant in the mid-lactation (cycles 4-6) high-producing dairy cattle, whereas the abundance was relatively lower during early lactation (Saxena et al., 2010; Li et al., 2018; Meyer et al., 2019). The results suggest that the role of the anaerobic rumen fungi in feed utilization and milk production may be improved with the maturation of the animals and stabilization of the ruminal ecosystem (Meyer et al., 2019; Williamson et al., 2022).

### 5.2 Mechanistic pathways that relate fungal activity to milk nutrient synthesis

Xue (Xue et al., 2020) found that cows with higher MPY (milk protein yield) showed higher levels of the key VFAs (volatile fatty acids), acetate and butyrate, than low-MPY animals. These metabolites are important precursors for biosynthesis of lipids in the mammary gland and are directly involved in milk fat synthesis (Xue et al., 2020). At the same time, the efficient degradation of lignocellulose by means of cellulosome-based enzyme systems of microorganisms increases the release of fermentable substrates and stimulates the growth of microorganisms in the rumen (Xue et al., 2020). Microbial biomass increases and more microbial protein passes into the small intestine to be digested and absorbed as amino acids. These amino acids are the primary substrates for the mammary epithelial cells for *de novo* synthesis of milk proteins. Xue (Xue et al., 2020) also proposed a central metabolism model linking rumen fermentation and milk production. Microbes degrade dietary fiber along this pathway to pyruvate as a central intermediate, which is then converted to volatile fatty acids (VFAs) via different biochemical pathways, where acetate and butyrate are generated through acetyl-CoA and propionate is mainly produced by the succinate pathway (Xue et al., 2020; Wilken et al., 2021; Jyothi et al., 2024; Deng et al., 2025). Anaerobic rumen fungi (ARF) are the first colonizers of lignocellulosic feed and induce mechanical disruption and enzymatic hydrolysis of plant biomass. This activity renders the substrate more accessible for subsequent bacterial fermentation and thus ARF are positioned as upstream facilitators of the fermentation cascade. Furthermore, Badhan (Badhan et al., 2025) showed that fungal metabolism during cellulose degradation produces hydrogen (H_2_) that influences ruminal redox balance and interspecies hydrogen transfer. This process is involved in the regulation of the acetate to propionate ratio, an important parameter affecting milk fat synthesis and overall energy partitioning in the dairy animal (Xue et al., 2020; Badhan et al., 2025). To move from correlation to causation, the use of integrative multi-omics approaches (Badhan et al., 2025), such as metagenomics, metabolomics and host serum profiling is essential (Xue et al., 2020; Badhan et al., 2025) as explained in **Table 1**. Such frameworks may offer mechanistic insights into how anaerobic rumen fungi affect host metabolism and production traits. Microbiome-aware animal selection is a promising frontier in the field of precision dairy breeding with great future potential.

**Table 1:** Representative studies linking anaerobic rumen fungi (mycobiome) with milk productivity traits in ruminants.

Host species	Methodology	Key fungal taxa identified	Productivity	Traits affected	Limitations	Study (year)
Cattle	Culture + molecular characterization	Neocallimastigomycota (*Neocallimastix*, *Piromyces*)	Demonstrated strong fibrolytic potential of anaerobic rumen fungi; enhanced fiber degradation efficiency	Indirect effect on milk yield via improved digestibility	Mechanistic; limited direct production data	2019 (Gruninger et al., 2019)
Cattle (rumen isolates)	Comparative genomics	Anaerobic fungal consortia	Identified extensive CAZyme repertoire; fungi possess superior biomass-degrading capability	Feed efficiency → potential increase in milk yield	No direct animal performance data	2018 (Cheng et al., 2018)
Dairy cattle	16S + ITS amplicon sequencing	*Neocallimastix californiae*, *Orpinomyces*	Positive correlation between fungal abundance and milk fat and protein traits	Milk fat %, milk protein %	Correlative; lacks causal validation	2022 (Agustina et al., 2022)
Global ruminant dataset	High-throughput sequencing	Diverse Neocallimastigomycota taxa	Diet-driven shifts in fungal communities; forage diets increased fungal abundance	Feed efficiency → milk yield potential	No direct milk trait measurements	2015 (Henderson et al., 2015)
Sheep/cattle	Amplicon sequencing (multi-domain)	Mixed anaerobic fungal taxa	Co-occurrence patterns indicate fungi–bacteria synergy in fiber degradation	Indirect effects on productivity	Limited fungal resolution (early ITS methods)	2013 (Koester et al., 2023)
Cattle	Microbiome profiling	Fibrolytic fungal populations	Higher fungal abundance associated with improved nutrient digestibility	Milk yield (indirect)	Lacks species-level fungal data	2013 (Morgavi et al., 2020)
Ruminants (review + experimental)	Ecosystem-level analysis	Anaerobic rumen fungi (general)	Highlighted role of fungi in hydrogen production and fermentation balance	Fermentation efficiency → productivity	Conceptual; not trait-specific	2017 (Haitjema et al., 2017)
Fungal isolates	Genomics + enzymatic profiling	Multiple anaerobic fungal genera	Discovery of fungal cellulosome-like complexes enhancing lignocellulose degradation	Feed utilization → milk output potential	*In vitro* focus	2017 (Hassan et al., 2020; Yu et al., 2025)

### 5.3 Species-specific insights: cattle vs buffalo from omics-based studies

Apparent differences in rumen fermentation kinetics were observed by Iqbal (Iqbal et al., 2018) in a comparative study of water buffalo (*Bubalus bubalis*) and Jersey cows under same feeding and management conditions (Iqbal et al., 2018). Buffalo had significantly higher concentrations of total volatile fatty acids (VFAs) (including acetate and propionate) and higher abundances of rumen bacteria, protozoa, fungi and important fibrolytic species such as *Fibrobacter succinogenes* (Iqbal et al., 2018). In conclusion, these parameters collectively indicate a better fiber utilization and improved fermentative efficiency. Interestingly the higher levels of acetate in buffalo correlate with their generally higher milk fat content compared with Holstein cattle (Iqbal et al., 2018). Physiologically, the buffalo ruminated ~50% longer with a ~30% slower particulate passage rate, conditions favoring longer microbial colonization, especially by anaerobic rumen fungi, and more efficient lignocellulose degradation (Iqbal et al., 2018). Recent progress in quantitative multi-omics approaches has provided strong evidence for the association of rumen function with milk production traits. A recent integration analysis indicated that rumen microbial composition (17.81%), functional pathways (21.56%), rumen metabolites (29.76%) and serum metabolites (26.78%) were able to explain the variation of milk protein yield, with a total explanatory power of around 47% (Kumar et al., 2015; Iqbal et al., 2018; Ji et al., 2022). Further, ITS amplicon sequencing at different stages of lactation (2 cycles, 6 time points) identified the genus *Caecomyces* as a potential biomarker associated with high efficiency animals in the second cycle of lactation (Kumar et al., 2015; Iqbal et al., 2018; Cox et al., 2021; Ji et al., 2022). However, a major limitation of these analyses is that the fungal and archaeal communities display relatively weak diversity and abundance signals (Iqbal et al., 2018; Ji et al., 2022). To address this, datasets are often pooled to increase statistical power, but this can result in a loss of taxonomic resolution and obscure finer-scale ecological and functional differences among these groups.

## 6 Methods to study the rumen mycobiome: critical comparison of approaches

The rumen microbiome has been of great interest because of its essential role in regulating the digestion and fermentation of feed and supplying most of the energy, nutrients, and metabolic precursors required for the production of ruminant-derived products (Xue et al., 2022; Yu et al., 2024b). Therefore, various methodological approaches have been employed to investigate the diversity, abundance and functional roles of fungi in the rumen ecosystem.

### 6.1 Culture-based approaches

Molecular methods are now available, but culture-based approaches still underpin the study of anaerobic rumen fungi, not least because they enable detailed phenotypic and enzymatic characterization (Kovács et al., 2025). These approaches are particularly useful for the characterization of fibrolytic enzyme systems, such as cellulases and xylanases, that are involved in the degradation of lignocellulose (Agustina et al., 2022; Costa-Roura et al., 2022; Kovács et al., 2025). These techniques have their advantages, but culture-dependent techniques have major limitations (Agustina et al., 2022; Costa-Roura et al., 2022). Many of the anaerobic fungal taxa show strong culturing biases and are recalcitrant to *in vitro* cultivation (Sirohi et al., 2013; Wei et al., 2016). In addition, these techniques are laborious, require strictly anaerobic conditions and special growth media, and are time-consuming and technically demanding (Sirohi et al., 2013; Wei et al., 2016). Therefore, culture-based approaches are not suitable for large-scale or longitudinal studies to characterize the full diversity and dynamics of rumen fungal communities (Sirohi et al., 2013; Wei et al., 2016).

Moreover, culture-based approaches remain a crucial methodology for isolating, classifying, and evaluating the functional capabilities of anaerobic rumen fungi. Studying live cultures allows researchers to observe exact phenotypic traits, measure enzymatic timelines, and test practical applications in ruminant nutrition (Sirohi et al., 2013; Wei et al., 2016). Using targeted culture methods from the rumen of cattle fed high-fiber diets, researchers successfully isolated a total of 20 distinct fungal cultures, and phenotypic characterization of these cultures revealed that all 20 isolates exhibited polycentric growth patterns. Unlike monocentric fungi that develop a single reproductive center, polycentric fungi form complex, extensive thalli with multiple centers of reproduction and branched rhizoidal networks capable of deeply penetrating plant tissues (Sirohi et al., 2013; Wei et al., 2016). Based on these morphological traits and subsequent characterization, the isolates were classified into two specific genera within the phylum *Neocallimastigomycota*: *Orpinomyces* and *Anaeromyces* (Sirohi et al., 2013; Wei et al., 2016). Furthermore, culturing these fungi *in vitro* allowed for the precise profiling of their extracellular, plant-cell-wall-degrading enzymes (Sirohi et al., 2013; Wei et al., 2016). Ultimately, culture-based testing of ARF established that *Orpinomyces* spp. outperform *Anaeromyces* spp. not only in pure enzyme yield but also as more effective physical fiber degraders (Sirohi et al., 2013; Wei et al., 2016). This highlights their potential value as direct-fed microbial additives or probiotics to maximize the utilization of low-quality, high-fiber crop residues in livestock (Sirohi et al., 2013; Wei et al., 2016).

### 6.2 Sequencing and quantitative PCR-based targeted quantification

The internal transcribed spacer (ITS) is the gold standard marker for identification of fungi and analysis of their diversity (Swift et al., 2021) and can be used to efficiently resolve the taxonomy of anaerobic rumen fungi at the molecular level. But a large fraction of anaerobic rumen fungi (ARF) are uncultured and therefore conventional taxonomic characterization is hampered. To overcome this constraint, integrative approaches combining the ITS-based profiling with the transcriptomic data have been increasingly utilized to achieve higher resolution in phylogenetic relationships and functional potential. The large subunit (LSU) rRNA gene is often recommended for ecological and community-level investigations because of its taxonomic stability. ITS and LSU data sets can be used synergistically to improve phylogenetic inference (Cox et al., 2021). Researchers (Gruninger et al., 2019) have performed a comprehensive global amplicon-based survey of the rumen ARF mycobiome. The survey comprised 206 samples from 15 host species from 15 countries on 6 continents. The study revealed striking diversity, identifying 81 of the 88 currently recognized ARF genera or candidate genera (Denman et al., 2008; Fouts et al., 2012). This broad diversity notwithstanding, only a few genera were consistently dominant (each >4% of relative abundance), including *Neocallimastix*, *Orpinomyces*, *Caecomyces*, *Cyllamyces*, NY9, and *Piromyces* (Denman et al., 2008; Fouts et al., 2012). Diversity patterns were different among host taxa, with higher ARF diversity in *Antilocapridae* and *Cervidae* than in *Bovidae*, suggesting host-specific ecological structuring (Denman et al., 2008; Fouts et al., 2012). Community structure analysis revealed a phylosymbiotic pattern, where host phylogeny had a strong impact on microbial composition, and host family and species explained a significant observed variance (Denman et al., 2008; Islam et al., 2025). Other factors, including diet composition, domestication status, and biogeography, also contributed to shaping ARF community structure (Denman et al., 2008; Fouts et al., 2012). However, overlap effects between these variables and sampling and geographic constraints limit the ability to precisely quantify individual contributions (Zeng et al., 2019).

Similarly, quantitative PCR (qPCR) allows quantification of the abundance of target microorganisms relative to the abundance of other microbes in the sample by quantification of the gene copy number, usually expressed as copies per milliliter of rumen fluid (Wirth et al., 2018; Xue et al., 2018). This approach generally targets conserved genetic markers, such as the ITS region for fungi and the 18S rRNA gene for eukaryotic microbes (Xue et al., 2018; Cortés-López et al., 2026). Due to this high sensitivity and specificity, especially when combined with species- or group-specific primers, qPCR is a useful tool to validate trends observed in high-throughput sequencing datasets. Leamkrajang (Leamkrajang et al., 2024) quantified total fungi, bacteria, protozoa, and selected bacterial taxa in rumen fluid from swamp buffalo, beef cattle, and goats by real-time PCR assays with targeted primers. Results showed that fungal abundance was significantly affected by dietary treatment. The average fungal load of swamp buffalo fed a roughage: concentrate ratio of 70:30 (22.97 × 10^6^ copies/mL) was significantly higher than the average fungal load of swamp buffalo fed a 100:0 roughage diet (6.71 × 10^6^ copies/mL) (Leamkrajang et al., 2024; Dhakal et al., 2025). These results further support the usefulness of qPCR as a quantitative validation tool that complements sequencing-based approaches. But qPCR has inherent limitations (Leamkrajang et al., 2024; Dhakal et al., 2025). It does not provide direct information about community composition other than the targeted groups and lacks taxonomic resolution at larger scales unless multiple specific assays are used (Leamkrajang et al., 2024; Dhakal et al., 2025). Furthermore, it does not give functional or metabolic information and needs to be combined with meta-omics approaches to interpret the ecosystem at a complete level.

Similarly, the shotgun metagenomics-based sequencing of the anaerobic rumen fungus *Orpinomyces* strain *C1A* revealed a large set of carbohydrate-active enzymes, including 357 glycosyl hydrolases, 92 carbohydrate esterases, and 24 pectate lyases, far exceeding the enzymatic diversity found in many aerobic fungi (Kovács et al., 2025). Xue (Xue et al., 2022) used an integrative meta-omics approach that combined metagenomics, metatranscriptomics, and metabolomics to holistically characterize the rumen ecosystem of dairy cattle. They compared nine high-efficiency and nine low-efficiency Holstein cows to identify microbiome-associated determinants of feed efficiency (Xue et al., 2018; Xue et al., 2022). The high efficiency group built more robust and highly interconnected microbial networks, suggesting better metabolic coordination and substrate utilization (Wirth et al., 2018; Zeng et al., 2019; Islam et al., 2025). Network and functional analyses identified important microbial taxa associated with improved carbohydrate metabolism, notably *Selenomonas* and the family *Succinivibrionaceae* (Denman et al., 2008; Fouts et al., 2012). At the metabolite level, six rumen-derived metabolites were identified as strong predictive biomarkers of feed efficiency, mainly related to carbohydrate metabolic pathways, including lactic acid, 5-aminovaleric acid, and 4-hydroxybutyrate (Wirth et al., 2018; Xue et al., 2018; Cortés-López et al., 2026). Mechanistically, nicotinic acid has also been reported to have an effect on rumen microbial activity mainly by increasing the production of butyrate, an important volatile fatty acid that stimulates proliferation and differentiation of rumen epithelium (Wirth et al., 2018; Xue et al., 2018; Islam et al., 2025; Cortés-López et al., 2026). Physiological development can be enhanced, and growth performance improved in dairy calves by modifying the rumen microbiome through diet during the preweaning period (Xue et al., 2018; Cortés-López et al., 2026).

### 6.3 Critical comparison of methodologies and integrative multi-omics: the way forward

Each methodological approach to interrogate the rumen mycobiome has its own analytical strengths but also its own technical and interpretative limitations (Gruninger et al., 2019). The internal transcribed spacer (ITS) amplicon sequencing is a widely used method to characterize the composition of fungal communities and has been successfully used to identify correlations between the composition of the mycobiome and phenotypic traits, such as milk yield (Henderson et al., 2015; Koester et al., 2023). However, its reliability can be affected by primer bias and incomplete coverage and annotation of fungal reference databases, which may decrease taxonomic resolution and comparability between studies (Haitjema et al., 2017; Morgavi et al., 2020). Shotgun metagenomics is able to overcome many of these limitations by characterizing taxonomic diversity and functional potential at the same time (Haitjema et al., 2017). Meta-transcriptomics expands this framework by providing information on gene expression at the sampling site (i.e., direct evidence for metabolically active pathways, e.g., fungal fibrolytic activity, during specific physiological states, e.g., feeding, lactation) (Haitjema et al., 2017). The main drawback to this approach is that RNA is labile, and sample handling and preservation need to be stringent to avoid degradation (Hassan et al., 2020). Finally, metabolomics, with platforms such as GC–MS or NMR, enables quantification of end products of microbial metabolism, including key volatile fatty acids (VFAs) such as acetate, propionate, and butyrate (Yu et al., 2025). These metabolites are direct biochemical indicators of rumen fermentation patterns and are closely correlated with host physiological outputs, such as milk fat and lactose synthesis (Yu et al., 2025).

**Figure 2:**
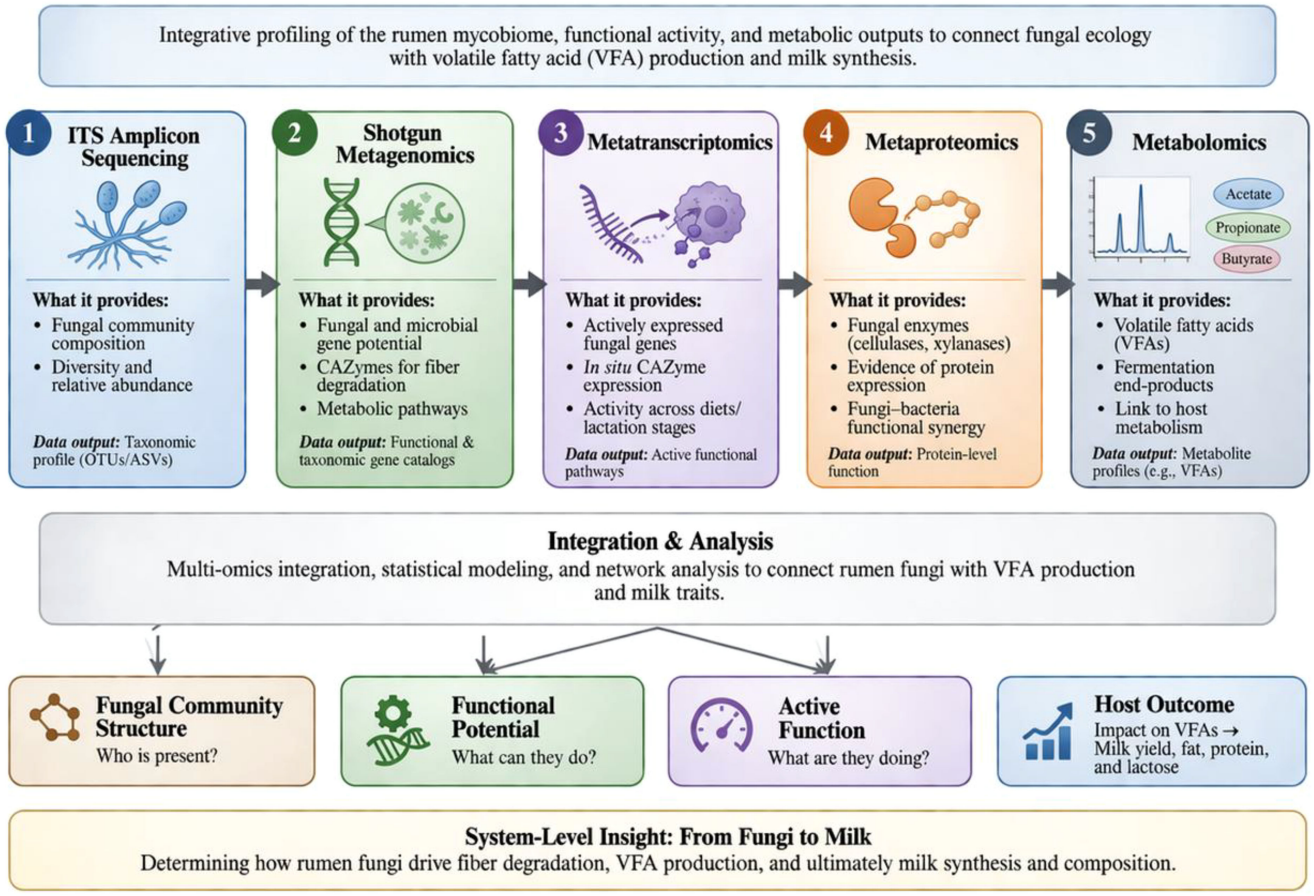
Linking rumen fungal community structure and activity to fiber degradation, VFA production, and milk synthesis using a multi-omics approach by integration of ITS amplicon sequencing, metagenomics, meta-transcriptomics, meta-proteomics, and metabolomics may provide systems-level insight into fungal functions.

The most complete resolution of the fungal contributions to rumen fermentation comes from an integrated multi-omics framework that combines ITS amplicon sequencing for community profiling, shotgun metagenomics for functional gene discovery, and meta-transcriptomics and meta-proteomics for validation of actively expressed pathways (Haitjema et al., 2017; Morgavi et al., 2020). This multi-layered approach allows for going from descriptive ecology to mechanistic understanding by linking microbial identity with functional activity and metabolic output (Yu et al., 2025). There is a need for systems-level integration of multi-omics data to develop predictive models (Tripathi et al., 2007) for feed efficiency, nutrient utilization, and methane emissions as explained in **Figure 2**. Researchers (Kazda et al., 2014; Lin et al., 2022; Dhakal et al., 2025) have also introduced the concept of “multi-omics rumen microbiome nutriomics” as a comprehensive framework to capture all microbial, nutritional, and host-driven influences (Hassan et al., 2020; Yu et al., 2025). Together, these studies come to a central conclusion: to move beyond correlative associations to establish causal relationships governing rumen function and animal performance, it is necessary to perform integrative analysis across omics layers along with metadata regarding diet composition, host genotype, and production traits (Xue et al., 2022; Yu et al., 2024b).

### 6.4 Key methodological gaps and recommendations

Although important progress has been made in research on the rumen mycobiome, there remain significant methodological and infrastructural barriers to a complete mechanistic understanding of the contribution of fungi to ruminant productivity (Gruninger et al., 2019). The major limitation is the lack of complete and well-curated fungal reference databases, especially for obligate anaerobic taxa belonging to the phylum *Neocallimastigomycota* (Haitjema et al., 2017; Hassan et al., 2020; Morgavi et al., 2020; Xue et al., 2022; Yu et al., 2024b; Yu et al., 2025). This limitation reduces the accuracy of taxonomic assignment in ITS-based amplicon sequencing and limits comparability among studies (Haitjema et al., 2017; Hassan et al., 2020; Morgavi et al., 2020; Xue et al., 2022; Yu et al., 2024b; Yu et al., 2025). The design of the primers is another source of bias. Existing ITS-targeting primer sets do not span the entire phylogenetic breadth of anaerobic rumen fungi and hence do not represent some genera (Hassan et al., 2020; Yu et al., 2025). Therefore, it is important to design and validate broad-spectrum fungi-specific PCR primers with high coverage and low amplification bias for reliable community profiling (Yu et al., 2024b).

Also, methodological harmonization is important. Variations in sampling protocols (time relative to feeding, rumen fraction sampled, i.e. solid vs. liquid associated microbiota and dietary context) introduce significant confounding (Xue et al., 2022). Harmonized sampling and metadata reporting frameworks would allow more robust comparative analyses and meta-studies. Researchers (Thongbunrod and Chaiprasert, 2026) have pointed out that targeted whole-genome sequencing, particularly for uncultured or poorly characterized species of *Neocallimastigomycota*, is a crucial priority to improve the genomic representation of anaerobic rumen fungi. These efforts would largely improve functional annotation pipelines and the interpretation of multi-omics data. Correlations between microbial profiles and host phenotypic traits including milk fat percentage, protein yield, feed conversion efficiency and methane emissions, open a route to predictive, microbiome-guided livestock management (Xue et al., 2022; Yu et al., 2024b; Thongbunrod and Chaiprasert, 2026). This will require concerted investment in high-quality comparative genomic and functional reference databases, standardized and reproducible sampling protocols, and longitudinal, multi-omics studies that follow fungal dynamics throughout the entire lactation cycle. These advances will support the development of next-generation microbiome-driven strategies for optimization of ruminant production systems (Henderson et al., 2015; Haitjema et al., 2017; Gruninger et al., 2019; Hassan et al., 2020; Morgavi et al., 2020; Xue et al., 2022; Koester et al., 2023; Yu et al., 2024b; Yu et al., 2025; Thongbunrod and Chaiprasert, 2026).

## 7 Manipulation of gut fungi for improved dairy productivity

Dietary manipulation is the most achievable and practical way to improve rumen fungal population (Tripathi et al., 2007; Elghandour et al., 2020; Yücel and Ekinci, 2022). Diets high in structural carbohydrates, especially cellulose and hemicellulose, result in longer rumen retention times, which favor colonization and activity of ARF (Tripathi et al., 2007; Yücel and Ekinci, 2022). These conditions increase the fibrolytic efficiency and enhance the overall feed utilization (Elghandour et al., 2020). However, nutritional interventions to reduce enteric methane emissions involve important trade-offs. Methane-mitigating compounds added to ruminant diets have been demonstrated to decrease methane production by 30–80% (Yücel and Ekinci, 2022). However, these strategies can unintentionally interfere with hydrogen metabolism, a critical driver of ARF activity 32]. The limitation in hydrogen supply can influence fungal fermentation processes and lead to inconsistent results for feed efficiency in different research studies (Tripathi et al., 2007; Yücel and Ekinci, 2022). The main problem, therefore, is to optimize dietary formulations that maintain hydrogen flux at a level that supports fungal metabolism, but have sufficient fiber to sustain fibrolysis (Tripathi et al., 2007; Yücel and Ekinci, 2022).

### 7.1 Rationale for targeting the rumen mycobiome

Anaerobic rumen fungi (ARF) can fibrolyse by mechanical means and by enzymes. ARF are characterized by extensive rhizoidal networks or, in some taxa, bulbous attachment structures for the structural invasion of plant tissues in some monocentric and polycentric members of *Neocallimastigomycota* (Swift et al., 2021; Hanafy et al., 2022). The rhizoids physically break down the integrity of the plant cell walls and thus increase the accessibility of the substrate. Moreover, the penetration of rhizoids may help to loosen the lignin-hemicellulose linkages and thus improve the degradability of lignocellulosic biomass (Hartinger and Zebeli, 2021; Hanafy et al., 2022). Targeted modulation of fungal populations represents a strategic opportunity to improve fiber digestibility, optimize volatile fatty acid (VFA) production, especially acetate, which is strongly associated with milk fat synthesis by improving feed conversion efficiency, and ultimately increase milk yield and compositional quality (Paul et al., 2018; Matthews et al., 2019; Hartinger and Zebeli, 2021). Anaerobic rumen fungi tend to be more ecologically stable under high fiber feeding regimes as opposed to many bacterial populations that are highly sensitive to sudden dietary shifts (Paul et al., 2018; Matthews et al., 2019; Hartinger and Zebeli, 2021). This inherent robustness, together with their strong fibrolytic potential, renders the rumen mycobiome a promising candidate for precision nutrition and microbiome engineering interventions to enhance ruminant productivity (Faniyi et al., 2019; Soltan and Patra, 2021).

### 7.2 Dietary interventions to modulate fungal populations

Several studies have shown that anaerobic rumen fungi (ARF) are more abundant in forage-based diets compared to concentrate-based feeding systems (Edwards et al., 2017). This response is mechanistically based on the specialized fibrolytic ability of ARF, which initiates the degradation of plant biomass using physical and enzymatic methods (Tedeschi et al., 2023; Dhillon et al., 2026). The invasive rhizoids of ARF breach the plant cell walls, weakening structural integrity and increasing substrate availability for microbial colonization (Tedeschi et al., 2023; Dhillon et al., 2026). ARFs secrete simultaneously a series of extracellular hydrolytic enzymes, including cellulases, hemicellulases, pectinases, and esterases, which act together to degrade complex lignocellulosic matrices (Dhillon et al., 2026). These sugars are then fermented via fungal fermentative pathways to primary end products, including formate, acetate, ethanol, lactate, CO_2,_ and H_2_ (Dhillon et al., 2026). This integrated process of mechanical and biochemical destruction is fundamental in order to improve the utilization of fiber and to control subsequent rumen fermentation processes (Tedeschi et al., 2023; Dhillon et al., 2026).

Moreover, type and processing of forage strongly affect the composition and activity of the rumen mycobiome (Faniyi et al., 2019; Dhillon et al., 2026). It is known that primary substrates, such as coarse, lignified forages, selectively stimulate fungal colonization due to their high structural complexity and larger particle size (Swift et al., 2021; Dhillon et al., 2026). This is mainly due to the unusual biology of anaerobic rumen fungi with invasive rhizoid systems adapted to penetrate hard plant tissues (Dhillon et al., 2026). Unlike bacteria, which are predominantly surface feeders, these fungi can physically penetrate the plant cuticle and lignified vascular bundles to form microfractures or “pores” within the plant matrix (Faniyi et al., 2019; Dhillon et al., 2026). This mechanical disruption increases fungi’s access to structural carbohydrates and the surface area for secondary colonization by fibrolytic bacteria, thus aiding the synergistic degradation of lignocellulosic material (Faniyi et al., 2019; Swift et al., 2021; Dhillon et al., 2026). On the contrary, plant biomass physicochemical properties, and hence its microbial degradability, can be greatly altered by forage processing techniques such as steam explosion. Steam explosion, an environmentally friendly pretreatment, disrupts the lignin-carbohydrate complexes, thus increasing the accessibility of cellulose and hemicellulose to enzymatic hydrolysis (Dhillon et al., 2026). Steam treatment of forages such as corn stover has been shown to improve *in vitro* ruminal fermentation and change fermentation patterns, indicating an increase in substrate availability for microbial degradation (Ji et al., 2022).

### 7.3 Probiotic approaches: fungal inoculation

The use of anaerobic rumen fungi (ARF) as direct-fed microbials (DFMs) offers a promising, but still understudied, approach in ruminant nutrition (Cheng et al., 2018; Hartinger and Zebeli, 2021; Hanafy et al., 2022). For example, the inclusion of ARF culture supernatant in grass silage has demonstrated a significant positive effect on *in situ* ruminal fiber degradability, indicating an improved breakdown of lignocellulosic substrates (Swift et al., 2021). Moreover, ARF inoculation during ensiling can modulate fermentation dynamics; in rice straw silage treated with anaerobic rumen fungi (10^6^ TFU/mL), a significant reduction in pH was observed after 30 days compared to untreated controls, indicating improved fermentation quality driven by increased accumulation of volatile fatty acids (VFAs) (Hanafy et al., 2022). There are, however, several practical limitations to the field-level application of ARF as DFMs. One of the major challenges is the delivery and establishment of viable fungal populations in the rumen. In experimental protocols, fresh fungal cultures are usually given by daily or weekly dosing either by incorporation in feed or by oral drenching (Cheng et al., 2018; Hartinger and Zebeli, 2021; Hanafy et al., 2022). Such approaches work well in a research setting, but are logistically infeasible for routine livestock management (Hartinger and Zebeli, 2021; Hanafy et al., 2022). Thus, there remains a critical need for the development of stable, scalable delivery systems (e.g., encapsulated formulations or anaerobically preserved inocula) to facilitate translation of ARF-based interventions into commercial practice (Matthews et al., 2019; Hanafy et al., 2022).

### 7.4 Rumen microbiome engineering

Recent advances in microbiome science have opened new opportunities to manipulate rumen ecosystems in a targeted way to achieve greater accuracy and predictability of fermentation outcomes and animal performance (Hartinger and Zebeli, 2021; Mizrahi et al., 2021; Hanafy et al., 2022; Tedeschi et al., 2023; Dhillon et al., 2026). A new approach is microbiome-informed selection and breeding, where animals with higher fungal abundance or increased fibrolytic activity are identified and incorporated into breeding programs to improve feed efficiency and productivity (Mizrahi et al., 2021; Jyothi et al., 2024; Dhillon et al., 2026; Tovar-Herrera et al., 2026). In addition, the generation of synthetic microbial communities, rationally designed microbial consortia containing anaerobic rumen fungi, bacteria, and methanogenic archaea, can be exploited to maximize the synergistic effects in the rumen, improving fiber degradation and stabilizing the fermentation pathways (Jyothi et al., 2024; Dhillon et al., 2026). At the same time, precision feeding approaches are gaining momentum, whereby diet formulations are tailored to the host’s current microbiome profile, and dynamically adjusted in response to lactation stage, physiological status, and production targets (Mizrahi et al., 2021; Deng et al., 2025). Importantly, species-specific differences should be taken into consideration while designing microbiome-based interventions, especially between *Bos taurus* (cattle) and *Bubalus bubalis* (buffalo) (Dhillon et al., 2026). Buffalo are generally reported as having a greater intrinsic fibrolytic capacity, a higher population of anaerobic rumen fungi on low-quality high fiber diets, and normally a higher milk fat content (Hartinger and Zebeli, 2021; Mizrahi et al., 2021; Hanafy et al., 2022; Dhillon et al., 2026). Cattle, however, are generally more responsive to dietary manipulation but also show greater variability in rumen function depending on breed, management, and nutritional inputs (Hartinger and Zebeli, 2021; Mizrahi et al., 2021; Hanafy et al., 2022; Dhillon et al., 2026). Hence, it is crucial to customize intervention strategies to species-specific rumen ecology to maximize the impact of microbiome engineering strategies and achieve optimal production outcomes.

Enhancing the rumen microbiota eukaryotic component may improve fungal activity and feed efficiency (Jyothi et al., 2024; Tovar-Herrera et al., 2026). The supplementation with live microorganisms is known to increase the relative abundance of the fibrolytic and ligninolytic groups, such as *Ascomycota* and *Neocallimastigomycota*, and in some conditions to stimulate the proliferation of *Chytridiomycota*, leading to a better utilization of feed components and reduced input costs (Mizrahi et al., 2021). The role of fungi in the control of the ruminal hydrogen flux is also of increasing interest, potentially affecting methane emissions indirectly through interaction with methanogenic archaea (Mizrahi et al., 2021; Dhillon et al., 2026). But more fungal activity could also produce more hydrogen, which could stimulate methanogenesis (Mizrahi et al., 2021). Hence, any intervention strategy needs to be carefully balanced to achieve productivity gains and environmental sustainability. Another important consideration is the potential for unintended shifts in the microbial equilibrium. There is emerging evidence for antagonistic interactions between anaerobic rumen fungi and rumen bacteria, with production of fungal metabolites with potential antibacterial properties and bacterial upregulation of efflux mechanisms in response (Mizrahi et al., 2021; Yu et al., 2025; Dhillon et al., 2026). Thus, most interventions currently in use are empirical and not optimized mechanistically.

### 7.5 Challenges and knowledge gaps in understanding the role of gut fungi in milk productivity

Almost all the studies of anaerobic rumen fungi have been correlative, relating fungal abundance or diversity to production traits like milk yield and composition, but not causally (Soliva et al., 2004). However, the dependence on associative analyses is not without its limitations as correlations are not causative and often confounded by interaction variables such as diet composition, host genetics, and architecture of bacterial and archaeal communities (Soliva et al., 2004). In addition, without controlled experimental validation, interpretation is difficult and adds uncertainty to directly attributing specific production outcomes to the activity of fungi, which limits the design of targeted and mechanism-based interventions (Gordon and Pesti, 1971; Soliva et al., 2004; Zhu et al., 2022). To fill these gaps, besides the use of advanced experimental systems such as the rumen simulation technique (RUSITEC) (Soliva et al., 2004) and gnotobiotic animal models (Gordon and Pesti, 1971), rigorous *in vivo* studies such as fungal enrichment or depletion experiments are needed. Moving from correlation to causation will require integrating multi-omics datasets and detailed phenotypic measurements (Zhu et al., 2022) as explained in **Table 2**. Another limitation of the field is the overemphasis on *Bos taurus* with a relative lack of research on *Bubalus bubalis* and indigenous breeds (Gordon and Pesti, 1971; Soliva et al., 2004; Zhu et al., 2022). This bias limits our understanding of species-specific fungal ecology and the applicability of findings to tropical, low-input, and smallholder production systems where buffalo and native breeds are often prevalent (Gordon and Pesti, 1971; Soliva et al., 2004; Zhu et al., 2022).

**Table 2:** Major challenges and knowledge gaps in linking anaerobic rumen fungi to milk productivity.

Challenges	Limitations	Causes	Research outcomes	Future directions
Taxonomic limitations	Poor annotation of anaerobic rumen fungi	Underrepresentation of Neocallimastigomycota in databases	Misclassification; underestimation of diversity	Develop curated ITS/genomic databases; expand reference genomes
Methodological bias	Primer bias in ITS sequencing	Non-universal primers; amplification inefficiency	Skewed community profiles; poor reproducibility	Standardize primers; adopt multi-marker approaches
Low detection sensitivity	Fungal underrepresentation in metagenomics	Dominance of bacterial DNA	Reduced resolution of fungal functional genes	Improve host/bacterial DNA depletion; increase sequencing depth
Culturing constraints	Difficulty in isolating anaerobic rumen fungi	Strict anaerobic growth requirements	Limited availability of reference strains	Develop advanced anaerobic culturing systems; culture collections
Functional uncertainty	Limited gene-function annotation	Poorly characterized fungal genomes	Weak linkage between taxa and function	Integrate metagenomics with transcriptomics/proteomics
Correlation vs causation gap	Lack of experimental validation	Predominance of observational studies	Uncertain role of fungi in milk traits	Conduct controlled *in vivo* trials; use RUSITEC models
Host species bias	Limited studies in buffalo and indigenous breeds	Focus on Bos taurus systems	Poor generalizability to tropical systems	Expand studies to Bubalus bubalis and native breeds
Microbial interaction complexity	Difficulty isolating fungal effects	Multi-kingdom interactions (bacteria, archaea, protozoa)	Oversimplified interpretations	Use network analysis and multi-omics integration
Temporal variability	Dynamic shifts in fungal populations	Diet, lactation stage, and feeding cycles	Inconsistent results across studies	Perform longitudinal and time-series studies
Spatial heterogeneity	Variation across rumen fractions	Solid vs liquid phase differences	Sampling bias; incomplete representation	Standardize sampling (fraction-specific analysis)
Data integration challenges	Difficulty combining multi-omics datasets	Lack of unified computational frameworks	Fragmented insights; underutilized data	Develop integrative bioinformatics pipelines; apply AI/ML tools
Translational limitations	Poor scalability to field conditions	Controlled lab vs variable farm environments	Limited practical application	Conduct field trials; develop scalable interventions
Conceptual bias	Bacteria-centric research paradigm	Historical focus on bacterial microbiome	Underestimation of fungal contributions	Adopt multi-kingdom microbiome frameworks
Environmental trade-offs	Uncertain impact on methane emissions	Increased hydrogen production by fungi	Potential increase in methanogenesis	Integrate fungal studies with methane mitigation research

## 8 Future perspectives: advancing rumen mycobiome research toward dairy productivity optimization

### 8.1 Methane mitigation

Rumen fermentation is estimated to contribute approximately 30% of anthropogenic methane emissions and is therefore an important target for climate mitigation strategies in livestock systems (Faniyi et al., 2019). Recent studies have focused on nutritional interventions that can modulate rumen fermentation and reduce enteric methane emissions. For example, the inclusion of freeze-dried *Sargassum mcclurei* at 2% of substrate in *in-vitro* studies significantly reduced methane production and increased crude protein degradability, suggesting its potential as a multifunctional feed additive to enhance fermentation efficiency and reduce greenhouse gas emissions (Li et al., 2024). Another major factor regulating rumen metabolism is dietary composition. It has been shown that the optimization of the energy-to-protein ratio enhances the efficiency of feed utilization and the total performance of the animals (Li et al., 2024). Additional experimental data have also shown that higher incubation temperatures could enhance ammonia nitrogen (NH3-N) and total volatile fatty acids (TVFAs) and reduce the abundance of bacteria related to the liquid phase, indicating temperature-dependent microbial activity and fermentation changes (Faniyi et al., 2019; Li et al., 2024). These results reveal the rumen microbial ecosystem’s sensitivity to physicochemical conditions and dietary inputs (Li et al., 2024). Such additives can reduce methane emissions, stabilize rumen pH, and reduce the risk of metabolic diseases such as acidosis and bloat.

### 8.2 Fungal feed additives

Fungal-derived feed additives, especially those from certain species such as *Saccharomyces cerevisiae* and *Aspergillus oryzae*, play a crucial role in improving animal nutrition and health (Cragg et al., 2015; Liang et al., 2020; Peng et al., 2021). These additives are rich in bioactive components such as exogenous enzymes, prebiotic oligosaccharides, and antioxidant compounds, which together enhance digestive efficiency, promote favorable gut microbiota, and support immune function (Cragg et al., 2015; Liang et al., 2020). Fungal supplements have the potential to improve nutrient digestibility and gut health, resulting in improved feed conversion efficiency and overall animal performance (Cragg et al., 2015; Liang et al., 2020; Peng et al., 2021). Also, some fungal additives have antimicrobial properties useful for the control of pathogenic microorganisms, improving animal welfare, and decreasing the incidence of diseases (Cragg et al., 2015; Liang et al., 2020; Peng et al., 2021). However, the efficacy of fungal feed additives is influenced by several factors such as strain specificity, dosage, diet composition, and host species, leading to variability of the observed results (Hess et al., 2020; Saye et al., 2021; Meili et al., 2024). This emphasizes the importance of further mechanistic studies to understand their modes of action and to develop standardized and targeted supplementation strategies (Cragg et al., 2015; Hess et al., 2020). For example, genera like *Candida*, *Talaromyces*, *Cyrenella*, and *Stilbella* were enriched in high-fat producing buffalo, while *Anaeromyces* was more abundant in their low-fat counterparts (Banks et al., 1990; Mathew et al., 2025). However, fungal feed additives have a huge potential to improve livestock productivity sustainably, with economic and environmental benefits, and to contribute to the advancement of modern animal husbandry practices (Hess et al., 2020; Saye et al., 2021; Meili et al., 2024). There is also an urgent need to expand fungal culture collections and to establish comprehensive genomic and functional reference databases to improve taxonomic resolution and functional annotation (Cragg et al., 2015; Liang et al., 2020; Peng et al., 2021). Feed additives play a crucial role in modulating rumen fermentation and improving the efficiency of nutrient utilization in ruminants (Liang et al., 2020; Meili et al., 2024). They influence the composition and activity of the rumen microbiota, thereby altering fermentation pathways and reducing hydrogen availability for methanogenesis (Cragg et al., 2015; Liang et al., 2020; Peng et al., 2021). Additives such as lipids, tannins, essential oils, nitrates, and 3-nitrooxypropanol (3-NOP) have been shown to suppress methanogenic archaea and redirect hydrogen toward alternative metabolic pathways, particularly propionate production (Hess et al., 2020; Saye et al., 2021; Meili et al., 2024). These changes not only reduce enteric methane emissions but also improve feed conversion efficiency and animal productivity (Cragg et al., 2015; Liang et al., 2020; Peng et al., 2021). Furthermore, the effectiveness of feed additives depends on their interaction with the host-associated rumen microbiome, which is influenced by both dietary and genetic factors. Therefore, feed additives represent a promising nutritional strategy for enhancing livestock sustainability while mitigating environmental impacts.

## 9 Conclusion

The rumen ecosystem is a highly coordinated multi-kingdom microbial network that underlies feed utilization and ultimately dairy productivity. Historically, the main focus of research has been bacteria and archaea, a growing body of evidence clearly indicates that anaerobic rumen fungi of the phylum *Neocallimastigomycota* are critical players in rumen function. The fungi can physically disrupt plant cell walls and secrete a wide range of fibrolytic enzymes that trigger the degradation of lignocellulose and enable subsequent microbial fermentation processes. This upstream role makes anaerobic rumen fungi central to substrate availability, volatile fatty acid (VFA) production, and feed conversion efficiency. Anaerobic rumen fungi can improve fibrolytic degradation and acetate production and indirectly influence milk fat synthesis. Their effect on fermentation efficiency also influences energy partitioning for lactation. Direct cause and effect relations between fungal communities and milk yield or composition are still poorly understood, but emerging multi-omics studies consistently point to links between fungal diversity, fermentation profiles, and production traits. These findings emphasize the importance of including the fungal ecology in current models of rumen function and dairy productivity.

Integrating anaerobic rumen fungi into an inclusive, multi-kingdom microbiome paradigm is an important step forward for dairy science and the future. Strategic dietary interventions, microbiome-informed feeding strategies, and perhaps the development of fungal-based additives to manipulate fungal populations are promising ways to improve feed efficiency and milk quality. Simultaneously, these methods should be assessed in terms of environmental sustainability, particularly regarding methane emissions and resource use. Overall, anaerobic rumen fungi are not only an accessory to the microbial community, but are key functional drivers of fiber degradation and metabolic efficiency. Incorporating fungal biology into rumen microbiome research will help achieve a more comprehensive understanding of host–microbe interactions and will promote the development of novel microbiome-based strategies for improving dairy productivity. These advances will be critical to meet the rising global demand for milk while providing sustainable and resilient livestock production systems.
